# Chromosome Deletion of 14q32.33 Detected by Array Comparative Genomic Hybridization in a Patient with Features of Dubowitz Syndrome

**DOI:** 10.1155/2011/306072

**Published:** 2011-09-28

**Authors:** Diana C. Darcy, Scott Rosenthal, Robert J. Wallerstein

**Affiliations:** South Bay Regional Genetics Center, Santa Clara Valley Medical Center, Suite 310, 751 S. Bascom Avenue, San Jose, CA 95128, USA

## Abstract

We report a 4-year-old girl of Mexican origins with a clinical diagnosis of Dubowitz syndrome who carries a de novo terminal deletion at the 14q32.33 locus identified by array comparative genomic hybridization (aCGH). Dubowitz syndrome is a rare condition characterized by a constellation of features including growth retardation, short stature, microcephaly, micrognathia, eczema, telecanthus, blepharophimosis, ptosis, epicanthal folds, broad nasal bridge, round-tipped nose, mild to moderate developmental delay, and high-pitched hoarse voice. This syndrome is thought to be autosomal recessive; however, the etiology has not been determined. This is the first report of this deletion in association with this phenotype; it is possible that this deletion may be causal for a Dubowitz phenocopy.

## 1. Introduction

Dubowitz syndrome was originally described in 1965 by Dubowitz [1], involving dwarfism with low birth weight, eczema, and distinctive facial appearance. Since 1965, approximately 200 cases have been reported [2–4]. Dubowitz syndrome is believed to follow a recessive mode of inheritance because of a number of case reports of siblings with Dubowitz syndrome with unaffected parents. 

Approximately, 10 cases of 14q32.3 terminal deletion have been reported, and although quite rare, a phenotype of the “14q32.3 deletion syndrome” has been established [5–7]. There are 23 specific clinical features attributed to Dubowitz syndrome; only two of which are specifically not part of the 14q32.3 deletion syndrome (2, 3 toe syndactyly and cytorchidism (see Table 1)). Furthermore there are 21 clinical features noted for the 14q32.3 deletion syndrome and five of those features have not been described in association with Dubowitz syndrome (high forehead, hypertelorism, lateral forehead hypertrichosis, single palmar crease, and clinodactyly (see Table 1)). There are 16 clinical features that overlap for both the 14q32.3 terminal deletion syndrome and Dubowitz syndrome.

Dubowitz syndrome is a multiple congenital anomalies syndrome with intellectual deficits and growth failure. Rarely do patients fit all of the clinical features of this syndrome. To date, Dubowitz syndrome has been described as a rare, autosomal recessive disease characterized by microcephaly, growth retardation, facial asymmetry, blepharophimosis, sparse hair and eyebrows, low-set ears, and mental retardation. In addition, other characteristics include a soft high-pitched voice, dental and craniofacial abnormalities, palate deformations, eczema, language difficulties, and an aversion to crowds. Significant phenotypic variability has been described. Because of a lack of array comparative genomic hybridization (aCGH) testing in other cases of Dubowitz, it is not possible to say whether our patient's deletion overlaps the cases previously described as the 14q32.3 terminal deletion syndrome. We believe that our patient with an array-detected 14q32.3 deletion has many features of Dubowitz syndrome.

## 2. Case Report

The proband is a female born to a 27-year-old gravida 3 para 2 mother by vaginal delivery which was induced at 33 weeks gestation because of intrauterine growth restriction (IUGR) and oligohydramnios. Her mother denies alcohol or drug use during pregnancy. The pregnancy was significant for IUGR since 20 weeks of gestation with a 3-4-week growth lag.

At birth, the proband was blue and floppy with no respiratory effort and required positive pressure ventilator assistance. Her APGAR scores were 5 at 1 minute and 7 at 5 minutes. Birth parameters were small for gestational age at 1477 g (10–25%), length 42 cm (25–50%), and head circumference 30 cm (25–50%). A wide fontanelle that was open from midforehead to occiput with continuity of posterior and anterior fontanelles, bilateral microphthalmia, small mouth with micrognathia, and a left low-set ear were noted. 7-dehydrocholesterol analysis was performed and was within normal limits. She remained in the NICU for 18 days and was then discharged to her parents. 

A review of the reported family medical history is non-contributory. There is no history of birth defects, developmental delays, mental retardation, or multiple miscarriages. The parents report no consanguinity. There are two older siblings without developmental concerns, birth defects, or dysmorphic features. 

**Figure 1:**
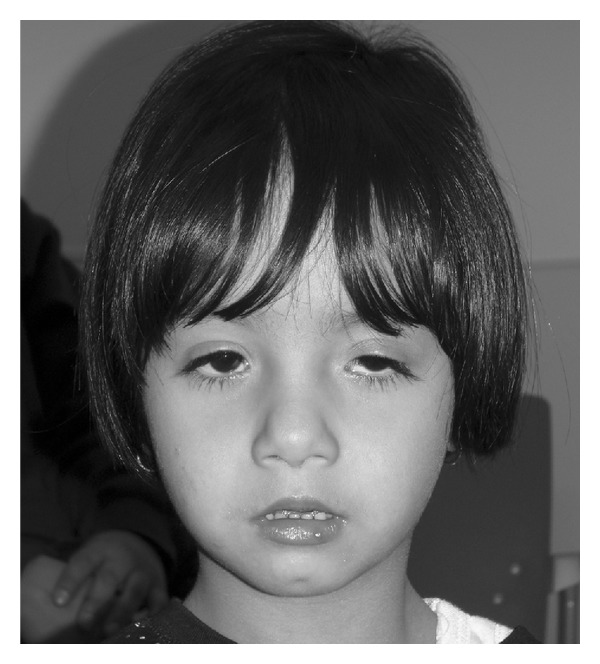
Patient at age of 2 years, 11 months. Used with permission.

**Figure 2:**
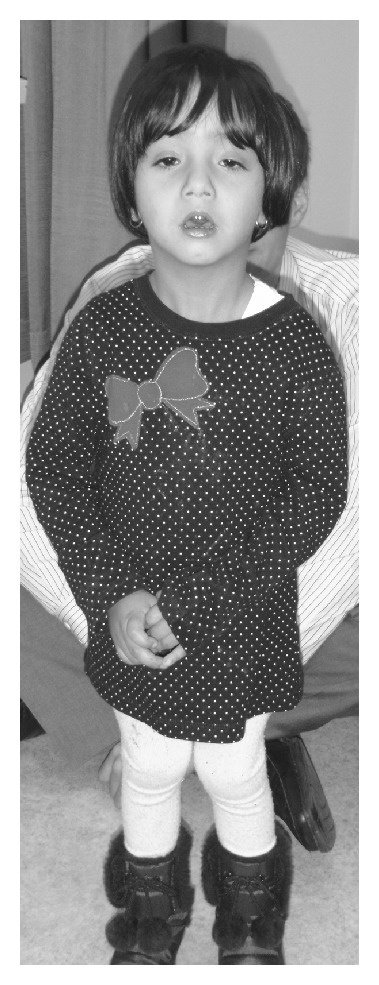
Patient at age of 2 years, 11 months. Used with permission.

**Figure 3:**
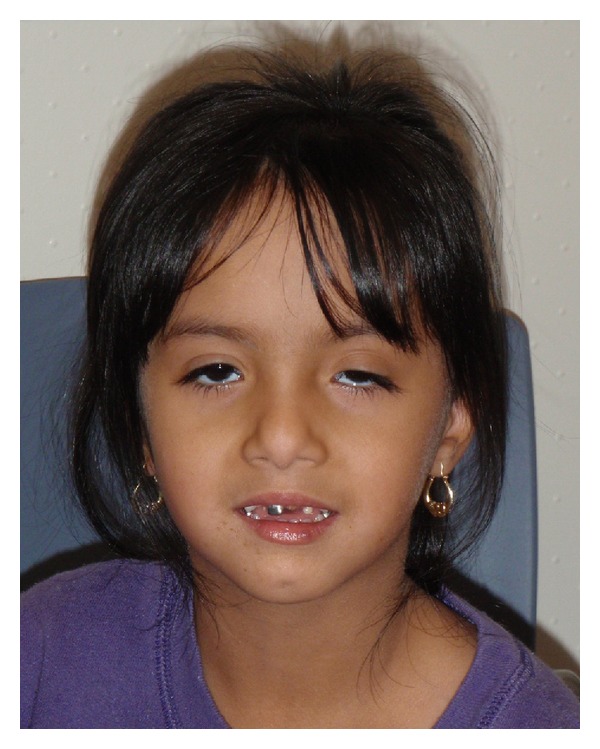
Patient at age of 4 years. Used with permission.

**Figure 4:**
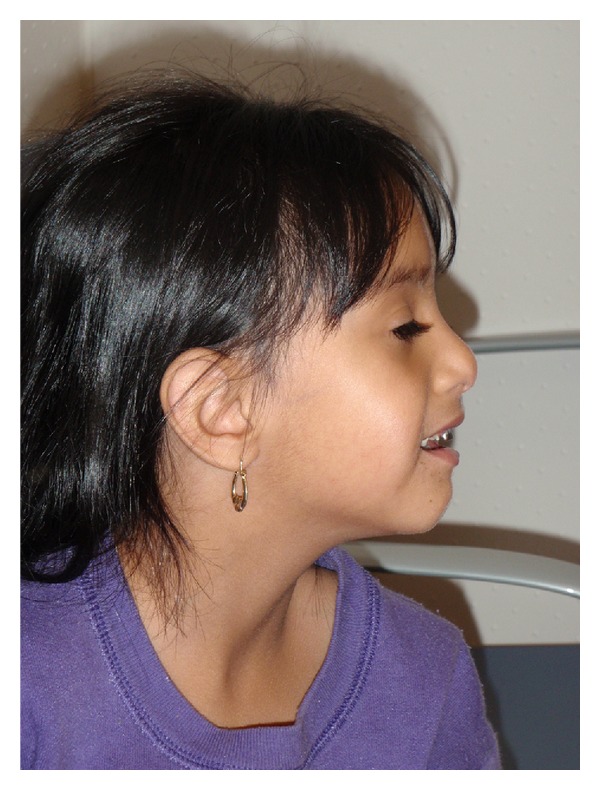
Patient at age of 4 years. Used with permission.

At 25 months of age, clinical features suggested a possible diagnosis of Dubowitz syndrome. The patient was noted to have an open-mouth habitus, ptosis of the left eye, downward slanting palpebral fissures, telecanthus, epicanthal folds, microcephaly (less than the 2nd centile), developmental delays and growth delays. By 48 months of age, the patient continued to have speech delay and reportedly knew many simple words and was just beginning to form 2-3-word phrases. The patient was attending a special education preschool to help with developmental skills. The patient continued to have significant difficulty gaining weight (approximately the 3rd percentile for her age group) despite high calorie nutritional supplementation (Figures 1, 2, 3, and 4). Her clinical features are listed in Table 1 along with a comparison of the majority of the features associated with Dubowitz syndrome and the 14q32 deletion syndrome. OF the previous 12 patients reported to have 14q32 deletion syndrome, the following craniofacial features are reported: broad philtrum (8 of 8), broad flat nasal bridge (7 of 8), telecanthus (8 of 10), hypotonia (8 of 10), high-arched palate (8 of 10) thin upper lip (4 of 5), blepharophimosis (6 of 8), pointed chin (5 of 9), malformed helices (4 of 7), small mouth (4 of 8), downward slanting palpebral fissures (4 of 8), and strabismus (4 of 9) [8]. Craniofacial features such as facial asymmetry, blepharophimosis, sparse hair and eyebrows, and low-set ears as well as other characteristics like growth-restriction and developmental disabilities are consistent with the gestalt of Dubowitz syndrome. 

**Figure 5:**
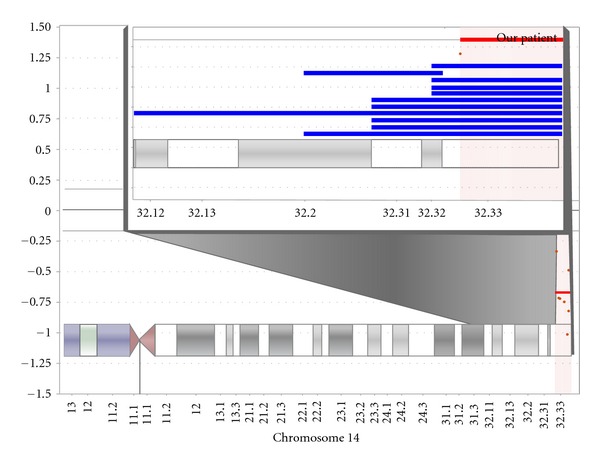
Relative chromosome breakpoints showing the location of the 2.77 Mb deletion at 14q32.33 to 14qter in our patient in relation to the previously described breakpoints for patients with the 14q32.3 deletion syndrome. The blue bars represent the approximate breakpoints and chromosome deletions in the other previously described patients of 14q32 deletion syndrome in [7]. This region includes 48 genes, 16 of which have a known phenotype.

Chromosome analysis showed a normal female karyotype of 46, XX at the 400–550 band level. Array comparative genomic hybridization (aCGH), performed in duplicate with 3141 BAC probes targeting multiple loci across all chromosomes revealed a genomic imbalance. The array exhibited a terminal deletion at 14q32.33 to 14qter between the BAC probes labeled CTD-2194E2 and RP11-47P23. This corresponds to genomic coordinates 103,572,825 to 106,339,477 according to hg18 annotation (Figure 5). 

Parental aCGH studies were subsequently performed which were normal, thus this deletion in the patient is presumably de novo. No other family members were tested.

## 3. Discussion

The differential diagnoses for Dubowitz often includes fetal alcohol syndrome and fetal alcohol spectrum disorders, Bloom syndrome, DiGeorge syndrome, Jacobsen syndrome, and Michels syndrome (oculopalatoskeletal syndrome). All of these syndromes were excluded for the patient. Bloom syndrome presents with pre- and postnatal growth deficiency, sun sensitivity, and telangiectatic hypo- and hyperpigmented skin. DiGeorge syndrome includes the presence of congenital heart defects, immunodeficiency, orofacial clefting, micrognathia, abnormal pinna, hypertelorism, and blunted nose. Jacobsen syndrome is identified by growth delays, developmental delays, trigonocephaly, strabismus, telecanthus, broad nasal bridge, and retrognathia. Michels syndrome is noted by blepharophimosis, blepharoptosis, and epicanthus inversus plus a developmental defect of the anterior segment of the eye as well as skeletal defects in the form of spina bifida occulta, craniosynostosis, cranial asymmetry, cleft lip and palate, hearing loss, and mild mental retardation. No specific skin findings or sun sensitivity in relationship with Bloom syndrome were noted for our patient. Additionally, the absence of congenital heart defects, orofacial clefting, skeletal anomalies, as well as trigonocephaly, or other forms of craniosynostosis makes a diagnosis of DiGeorge, Jacobsen, and Michels syndrome unlikely. 

There are forty-eight known genes in the region of interest; sixteen of which have been associated with specific clinical features (Table 2). Additionally, there are thirteen immunoglobulin genes in this region. Eczema, which is a major feature of Dubowitz syndrome, could be related to dysregulation of immunoglobulins. *CKBE*, a gene associated with brain type creatine kinase, is a necessary component of energy metabolism in the central nervous system. *HFM* and *MCOP1* are genes associated with hemifacial microsomia and isolated type 1 microphthalmia, respectively. Both of which are associated with craniofacial morphogenesis which could be linked to the Dubowitz phenotype. *SMALED*, autosomal dominant lower extremity spinal muscular atrophy, is also a part of the deleted region. We feel that this is a gene of interest due to hypotonia being a common feature in Dubowitz and chromosome 14q32 deletion syndromes.

Within the region of the deletion, the gene *JAG2* is present and is known to create a ligand for the receptor protein Notch1. *JAG1*, associated with the autosomal dominant Alagille syndrome, and *JAG2* are similar in structure to all of the currently identified Notch ligands [9, 10] and the Notch signaling pathway is a conserved intercellular signaling mechanism that is essential for proper embryonic development in numerous metazoan organisms. Additionally, mouse models have shown that Notch signaling mediated by Jag2 plays an essential role in limb, thymic, and craniofacial development [11]. The association of Notch1-induced skeletal deformations via osteoblast differentiation as well as *JAG1* and *JAG2* association with facial dysmorphism lend support that *JAG2* is a critical gene disrupted and may result in the proband's phenotype.

Dubowitz syndrome is a rare condition which is previously believed to be autosomal recessively inherited with no known diagnostic testing available. We believe that our patient provides an example of a chromosome abnormality with clinical features of Dubowitz syndrome. We suggest that the 2.77 Mb deletion at 14q32.33 to 14qter may shed light on the pathogenesis of Dubowitz syndrome in the future and bring a clearer understanding of the genes and loci involved in these phenotypes. Historically, chromosome deletions and rearrangements via chromosome analysis have aided the medical community in identifying candidate regions for disease identification. Deletions, like that seen in our patient, can identify candidate genes in autosomal recessive conditions by causing a knockout effect. At this time, we cannot determine whether the cases of Dubowitz-like syndrome are related to a chromosome deletion as a single causative factor or if the deletion would knock out one copy of the gene and there is another pathogenic mutation in the same gene on the homologous chromosome. Future studies may be warranted.

Testing other patients with Dubowitz using aCGH technology could reveal whether this is an isolated occurrence or common to patients with Dubowitz. *JAG2 *gene disruptions via gene deletions or point mutations may be resulted in Dubowitz syndrome or the phenotype previously described as the 14q32.3 deletion syndrome. This could also cause a contiguous gene syndrome effect resulting in the described phenotype. In addition, aCGH testing of those with 14q32 terminal deletion syndrome could help determine whether there is overlap between our patient's deletion and the previously reported deletions.

## Figures and Tables

**Table 1:** Comparison between the clinical features of Dubowitz syndrome, 14q.32 deletion syndrome, and the patient.

Feature	Dubowitz syndrome	14q.32 deletion syndrome	Patient
IUGR	+	+	+
Low birth weight	+	+	+
Microcephaly	+	+	+
Poor feeding	+	+	+
Postnatal growth retardation	+	+	+
High forehead	−	+	+
Ptosis	+	+	+
Blepharophimosis	+	+	+
Telecanthus	+	+	+
Epicanthal folds	+	+	+
Hypertelorism	−	+	−
Asymmetry in eye features	+	Not reported	+
Wide nasal bridge	+	+	+
Round tipped nose	+	+	+
Dysplastic low-set ears	+	+	+
Micrognathia	+	+	+
Palate anomalies	+	+High arched	−
Sparse, light-colored hair	+	Not reported	−
Lateral forehead hypertrichosis	−	+	−
Hypotonia	+	+	+
Mild or moderate developmental delay in 50%	+	+	+
Hyperactivity (in some)	+	Not reported	−
Eczema (~50% cases)	+	Not reported	−
High-pitched, hoarse voice	+	Not reported	+
Syndactyly 2nd and 3rd toes (in some)	+	−	−
Single palmar crease	−	+	−
Clinodactyly	−	+	−
Cryptorchidism, inguinal hernias, and hypospadias in some boys	+	−	−Patient is female

**Table 2:** A list of the 16 genes deleted with a known phenotype on chromosome 14 with the Online Mendelian Inheritance of Man (OMIM) annotation. We believe that CKBE, HFM, MCOP1, and SMALED (noted by asterisk) may be significant in the previously described 14q32 deletion syndrome.

Gene	OMIM annotation	Gene name
PHOBS	608251	Phobia, specific
IBGC1	213600	Basal ganglia calcification, idiopathic (Fahr's disease)
MNG1	138800	Multinodular goiter-1
CTAA1	115650	Catarct, anterior polar 1
CHDS4	608318	Coronary heart disease, susceptibility to, 4
CKBE*	123270	Creatine kinase, ectopic expression
GEVQ1	608875	Gene expression, variation in, quantitative trait locus on chr. 14
HFM*	164210	Hemifacial microsomia
MCOP1*	251600	Microphthalmia, isolated 1
SMALED*	158600	Spinal muscular atrophy, lower extremity, autosomal dominant
IGHR	144120	Immunoglobulin heavy chain regulator
XRCC3	600675	X-ray repair, complementing defective, repair in Chinese hamster cells-3
INF2	610982	Inverted forming 2
AKT1	164730	Murine thymoma viral (v-akt) oncogene homolog-1
IGHG2	147110	Constant region of heavy chain of IgG2
IGHM	147020	Constant region of heavy chain of IgM
